# COVID-19: A Comparative Study of Population Aggregation Patterns in the Central Urban Area of Tianjin, China

**DOI:** 10.3390/ijerph18042135

**Published:** 2021-02-22

**Authors:** Peng Zeng, Zongyao Sun, Yuqi Chen, Zhi Qiao, Liangwa Cai

**Affiliations:** 1 School of Architecture, Tianjin University, Tianjin 300272, China; urbanplan@tju.edu.cn (P.Z.); cailw@163.com (L.C.); 2 Tianjin University Research Institute of Architectural Design & Urban Planninng Co., Ltd, Tianjin 300350, China; YUaloha@tju.edu.cn; 3 School of Environmental Science and Engineering, Tianjin University, Tianjin 300350, China; qiaozhi@tju.edu.cn

**Keywords:** COVID-19, public-health resilience, ‘people-oriented’ concept, population agglomeration index (PAI) and population tidal index (PTI), dense urban area of China

## Abstract

When a public health emergency occurs, a potential sanitation threat will directly change local residents’ behavior patterns, especially in high-density urban areas. Their behavior pattern is typically transformed from demand-oriented to security-oriented. This is directly manifested as a differentiation in the population distribution. This study based on a typical area of high-density urban area in central Tianjin, China. We used Baidu heat map (BHM) data to calculate full-day and daytime/nighttime state population aggregation and employed a geographically weighted regression (GWR) model and Moran’s I to analyze pre-epidemic/epidemic population aggregation patterns and pre-epidemic/epidemic population flow features. We found that during the COVID-19 epidemic, the population distribution of the study area tended to be homogenous clearly and the density decreased obviously. Compared with the pre-epidemic period: residents’ demand for indoor activities increased (average correlation coefficient of the floor area ratio increased by 40.060%); traffic demand decreased (average correlation coefficient of the distance to a main road decreased by 272%); the intensity of the day-and-night population flow declined significantly (its extreme difference decreased by 53.608%); and the large-living-circle pattern of population distribution transformed to multiple small-living circles. This study identified different space utilization mechanisms during the pre-epidemic and epidemic periods. It conducted the minimum living security state of an epidemic-affected city to maintain the operation of a healthy city in the future.

## 1. Introduction

Under the concept of people-oriented urban design [1], the interaction between human behavior and urban material space is the most important dimension to study the regulation of urban processes [2,3,4,5,6]. Urban population distribution [7,8,9], like population activity density [10] and urban vitality distribution [11], is the dependent variable of urban spatial behavior that can be used to characterize the operational state of an urban area [12]. The population distribution and flow in a high-density urban area are the consequence of material space, functional space, and public policy. In recent years, researchers from different countries have begun to study the interactive relationships among population distribution and other factors, including urban function [13,14,15], urban spatial structure [16,17,18], and regional policy [19,20]. These studies have revealed many aspects of urban population distribution, but have concentrated on its static characteristics, which has limited value in actual daily life.

In recent years, many studies have considered the multi-period spatial population distribution patterns caused by differences in residents’ spatial behaviors. Li used the population density index (PDI), spatial correlation index (SCI) and ordinary least squares (OLS) model to investigate the spatial-temporal distribution and urban demographic structure in Xi’an, based on Baidu heat map (BHM) and points of interest (POI) data [21]. Shi et al. investigated the correlation between population density and the location of public facilities in Shanghai, using the data of 20 million 2G mobile phone records and the land use data of urban public service facilities [22]. These studies analyzed the daily spatial characteristics of urban populations using many different research methods. However, cities are complex giant systems that experience a range of different events. The response relationship between a fixed objective environment and population behavior patterns during particular events have not been fully explained by the above studies. Exploring the relationship between population distribution and spatial elements during specific events will be the main direction of future research in this field.

The outbreak of COVID-19 has changed residents’ behavior patterns and has also reshaped the urban function mode [23,24,25]. There is a significant relationship between public health and population distribution [26,27]. Most recent studies have focused on changes in inter-city population movements [28,29,30], but there have been few studies of the relationship between residents’ behavior changes and their spatial traits within a city. There have been no studies of how the solidified urban material space accommodates different patterns of residents’ behavior in an epidemic/pre-epidemic situation. This study considered the corresponding relationship between population spatial behavior patterns and physical space before and during a major public-health mergence. The study has attempted to improve urban public health resilience by comparing the changes of spatial demand in high-density urban areas before and during an epidemic.

Section 2 of this paper introduces the data and methods used in the study. Section 3 presents the population distribution and the characteristics of population flow via the population aggregation and population-tide features. A geographically weighted regression (GWR) model and Moran’s I were used to analyze the characteristics of population aggregation and the mechanisms influencing day-and-night tides before and during the epidemic period. Section 4 discusses urban spatial organizing mode under the perspective of public-health resilience and the shortcomings of the study, while Section 5 presents the main conclusions.

## 2. Materials and Methods

### 2.1. Study Area

**Figure 1:**
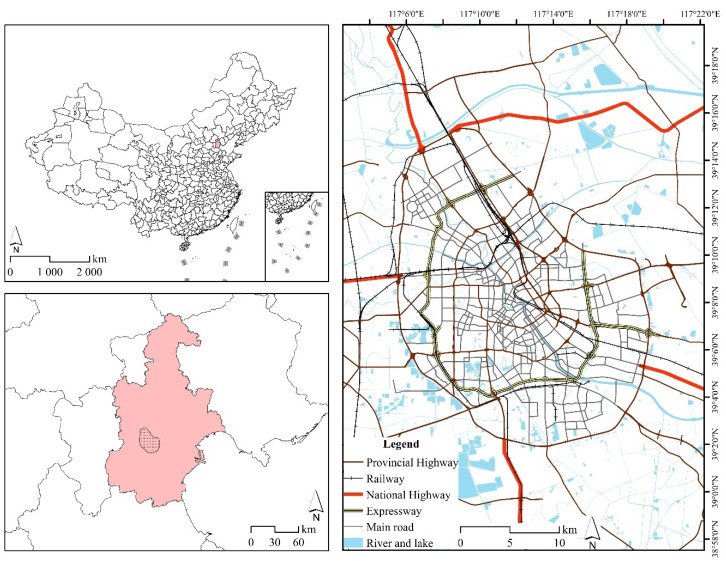
Study area.

This study focused on urban area within Tianjin Outer Ring Road (117°5′31′′–117°19′15′′ E, 39°1′54′′–39°15′22′′ N), which is the central area of political, cultural and economic activities in the city (Figure 1). It has a land area of 576.160 km^2^ (13.292% of Tianjin), and a population of 5.08 million people (39.543% of Tianjin, according to the Chinese Sixth Census). In this area, the average block size is 160,000 m^2^, and we used a grid with dimensions of 400 m × 400 m as the basic unit of analysis.

### 2.2. Data Sources

Tianjin had experienced four periods during the COVID-19 epidemic. The first was the discovery period (prior to 23 January 2020); the second was the control period (from 24 January to 10 February 2020); the third was a gradual resumption of activity period (from 11–29 February 2020) and finally there was a post-epidemic recovery period (from 1 March 2020 onwards). The gradual resumption period was a phase transformation between the suspension of urban activity and normal operation. It represented an operating state under the constraint of various control measures. Therefore, we selected 29 November 2019 and 27 February 2020 as the representative days of the urban area in the pre-epidemic and epidemic periods, respectively [10,31].

This study used BHM to illustrate the urban spatial demographic characteristics. Based on the geographic location data of mobile-phone users on a location-based services (LBS) platform, the BHM can visualize the spatial population aggregation through certain algorithms and reflect real-time population distributions [21,32]. We obtained the BHM data every half hour, and then conducted a projection conversion and reclassification to build population distribution dataset. Because seven colors represented seven population density levels, the original pictures were transformed from colors to 0–7 value tiff rasters [10,31].

In the GWR model, the impact factors were calculated by the data that were obtained from the Baidu open platform (http://lbsyun.baidu.com/ (accessed on 28 February 2020)) and Map World (http://tianditu.gov.cn (accessed on 13 June 2017)). The datasets included the Tianjin building footprint, main traffic network, urban subway stations, the area of urban blue/green spaces, and points of interests (POIs).

### 2.3. Methods

**Figure 2:**
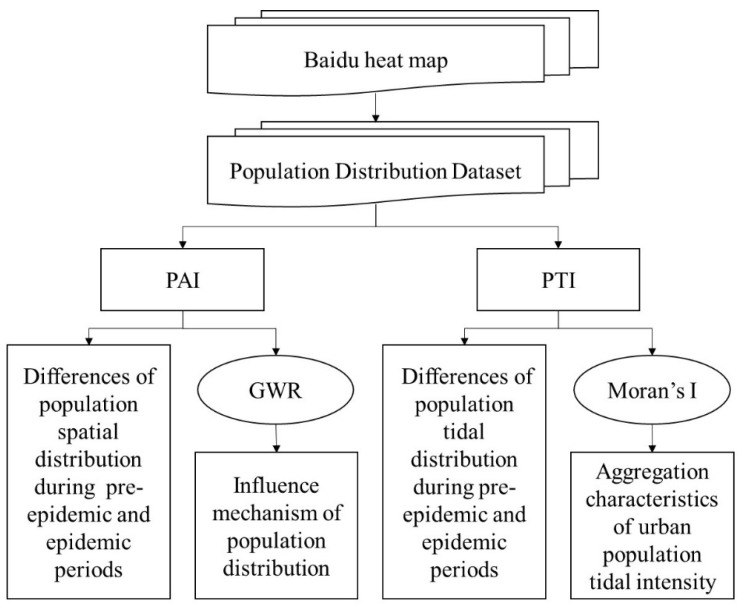
Technology roadmap.

In this study, the quantitative population indicators, population agglomeration index (PAI) and population tidal index (PTI), were constructed by the BHM, and analyzed by a GWR model and Moran’s I index, respectively. The technology roadmap is shown in Figure 2.

#### 2.3.1. The PAI and PTI

The PAI represents the population aggregation within an urban space. This study used the average population density in spatial units (400 m × 400 m) during active time (10:00 am–21:59 pm) as the indicator (Equation (1)). The PAI value range was 0–7; a high value represents a high population density. The PTI was calculated based on the PAI. It represents the difference between mean PAI in daytime (10:00 am–16:59 pm) and nighttime (17:00 pm–21:59 pm) (Equation (2)). The PTI ranges from −7 to 7, with a negative value indicating that the population gathering intensity is higher in the nighttime than in the daytime, and the day-and-night difference increases as the value decreases. A positive value indicates that the population gathering intensity is higher in the daytime than in the nighttime, and the day-and-night difference increases as the value increases:

(1) $$PAI_{t1\sim t2}=\frac{\sum_{t=t1}^{t2}\left(\frac{\sum_{g=1}^{7}HV_{g}\times HC_{g}}{\sum_{g=1}^{7}HC_{g}}\right)}{t2-t1}$$

where, $PAI_{t1\sim t2}$ represents the average PAI of spatial units during the *t*1–*t*2 period; $HV_{g}$ represents the score of the thermal color of g-grade, and $HC_{g}$ represents the number of grids of the g-grade:

(2) $$PTI=PAI_{10\sim 16}-PAI_{17\sim 21}$$

where, PTI is the PTI in Equation (2).

#### 2.3.2. The GWR Model

There are many factors influencing the urban population distribution, such as the quality of the built environment [33,34], public service conveniences [35,36], traffic accessibility [37,38,39], spatial ecological quality [40,41], and others [42,43]. There is spatial heterogeneity within each factor and traditional linear regression models have not been fitted well in previous studies [44,45], so we used a GWR model to figure out the population distribution mechanism. GWR is a revised spatial linear regression model [46,47,48]. When facing spatial heterogeneity, it can deal with the spatial relations among multiple variables and correctly analyze the spatial differences:

(3) $$y_{i}=\beta_{0}(u_{i},v_{i})+\sum_{j=1}^{k}\beta_{j}(u_{i},v_{i})x_{ij}+\varepsilon_{i}$$

where $y_{i}$ represents the induced variable; $\beta_{0}\left(u_{i},v_{i}\right)$ represents the constant of unit *i*; and $\beta_{j}\left(u_{i},v_{i}\right)$ is the regression coefficient of the *j*-th parameter of unit *i*, which reflects the spatial analysis of different parameters’ impact on unit *i*. The value of the coefficients indicates the strength of the correlation, with a positive or negative value representing a positive or negative correlation of the parameters and spatial positions respectively. $\left(u_{i},v_{i}\right)$ represents the coordinates of unit *i*; $x_{ij}$ represents the *j*-th value of unit *i*; $\varepsilon_{i}$ represents the random error; and *k* is the number of independent variables.

In the process of indexes selection, we first selected building density (BD), floor area ratio (FAR), distance to a subway station (DS), distance to a main road (DR), POI quantity (POI-Q), POI diversity (POI-D), and Proportion of blue and green space (BGR) to build an indicator system. POI-D was rejected by a collinear test in the OLS model (VIF > 10). Except POI-D, the other six indicators were standardized to form a collection of variables.

#### 2.3.3. Spatial Autocorrelation

The spatial autocorrelation analysis is assessing the dependence and heterogeneity of grids (400 m × 400 m) by the Moran’s I index [47,49]. The global Moran’s I indicates the overall distribution of data in a research area, while the local Moran’s I evaluates the similarity and differences of adjacent units. Both indexes range from −1 to 1, with a positive value indicating similarity and a negative value indicating difference. The degree of similarity and difference increases as the value increases. The Z-score and *p*-value represent levels of spatial association and their significance, respectively. The local Moran’s I value can be used to discriminate between four spatial types. The HH type represents a high value agglomeration, indicating that the partial mean is higher than the overall mean, while the LL type represents a low value agglomeration, indicating that the local average is lower than the overall average. The HL unit is a high value surrounded by low values, while the LH type is a low value surrounded by high values. In this study, HH and LL indicated aggregated population outflow units and aggregated population inflow units, respectively, while HL indicated local population outflow units and LH indicated local population inflows units:

(4) $$I=\frac{n}{S_{0}}\times \frac{\sum_{i=1}^{n}\sum_{j=1}^{n}\omega_{ij}•(x_{i}-\bar{X})(x_{j}-\bar{X})}{\sum_{i=1}^{n}{\left(x_{i}-\bar{X}\right)}^{2}}$$

(5) $$S_{0}=\sum_{i=1}^{n}\sum_{j=1}^{n}\omega_{ij}$$

where $x_{i}$ and $x_{j}$ are the attribute values of features *i* and *j*; $\bar{x}$ is the average of *n* cells’ attribute values; $\omega_{ij}$ is the spatial weight matrix. In this study the Queen’s Case (adjacency defined by mutual edge or point) was applied as the definition rules; and $S_{0}$ is a collection of spatial weights.

## 3. Results

According to the technology roadmap, the results were obtained from the perspective of population distribution and population tide characteristics during the pre-epidemic and epidemic periods.

### 3.1. Differences in the Population Distribution between the Pre-Epidemic and Epidemic

**Figure 3:**
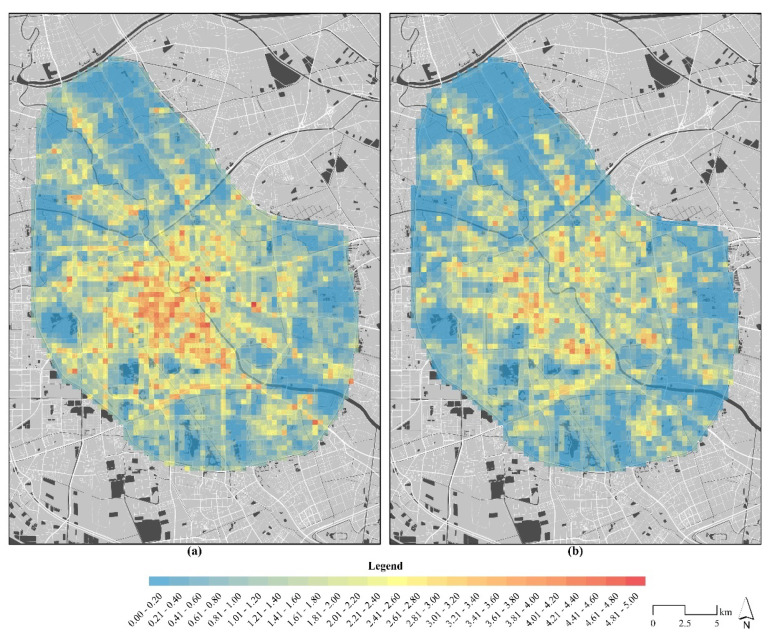
The population agglomeration index distribution during the pre-epidemic (**a**) and epidemic (**b**) periods.

The Moran’s I results showed the average PAI during the pre-epidemic period was 1.309, while during the epidemic the value was 1.065 (Figure 3). The intensity of pre-epidemic population aggregation was higher than during the epidemic. The pre-epidemic population aggregated in the central area around the Haihe River, which was presented spatially as a single core circle. The intensity around the middle of the Haihe River was higher than that in the east. There was a low aggregation phenomenon in the peripheral spaces that was apparent only near the main roads. In contrast, during the epidemic period, population aggregation decreased with the dissolution of the single core circle structure, while peripheral population aggregation increased. Homogenization became the main characteristic, with aggregation around the Haihe River and near the main roads decreasing significantly.

### 3.2. Differences in the Influences on the Urban Population Distribution between the Pre-Epidemic and Epidemic Periods

Table 1 and Table 2 present results of the GWR model explaining the distribution of the population during the pre-epidemic and epidemic periods, respectively. In both periods, the R^2^ value was higher than 0.75, and the model had strong explanatory power. In the results, MEAN represents the average coefficient, while STD, MIN, MAX indicate the standard deviation, maximum, and minimum, respectively. The area ratio of significance was the significant indicator, representing the proportion of significant units. The term + indicated the same relevance unit, while—was the opposite. During the pre-epidemic, the POI-Q had the biggest spatial influence area, with 93.752% significant units. The Area ratio of significance of the DS and the BGR was lowest at 13.829% and 12.774%, respectively. There were negative correlations for BD, DS, DR, and BGR, with DR presenting the largest correlation coefficient among the four indicators. The increase in the DR value corresponded to 0.305 units of PAI. The FAR and POI-Q were positive factors for population aggregation, with both having an average correlation coefficient of over 0.5, and the proportion of significant units reached 100%.

During the epidemic period, the POI-Q and FAR were significant in most spaces in the study area, at 83.921% and 71.091%, respectively (Table 2). Compared with the pre-epidemic period, the significance percentage of epidemic POI-Q decreased by about 10% and the interpretative ability of spatial functionality decreased significantly, while the explanatory power of FAR rose from 37.545% to 71.091%. The significant unit ratio of DS, DR and BGR was lower than 15%. Compared with pre-the epidemic period results, the proportion of significant DS units was basically stable. The DR decreased significantly from 69.092% (2nd) to 8.275% (6th), and the area of influence also shrank. The BGR decreased from 12.774% to 9.164%, with the restriction on the use of leisure space (e.g., parks and riverbanks) partially affecting the internal mechanism of population aggregation.

Comparing the positive and negative significant unit ratios, the FAR, DS, and BGR were clearly different. The significant ratio of DS changed from 81.928–18.072% to 34.362–65.638%, showing that some positive units changed into negative units, and the PAI decreased as the number of these changes increased. In the epidemic model, the FAR coefficient was the largest (0.669). The POI-Q coefficient reduced to 0.543, indicating that its influence on population flow was substantially weakened. Among the negative indexes, the decreases in BD and BGR indicated that their influence was enhanced. The influence of DR and DS decreased too.

Comparing the results of the two models, it was apparent that the demand for outdoor activities was greatly reduced during the epidemic period, resulting in an enhancement of the correlation and significance of the built-environmental indicators (BD, FAR), and a decline of the correlation and significance of the spatial functional index (POI-Q), the spatial quality index (GBR), the traffic access index (DS, DR). Because of the single-core structure in the city and the travel restrictions, the limited travel radius in the high-density area weakened the ability of traffic to reshape the population pattern, while spatial units were more strongly influenced by their adjacent units.

### 3.3. Differences of Population Tidal Distribution during Pre-Epidemic and Epidemic Periods

**Figure 4:**
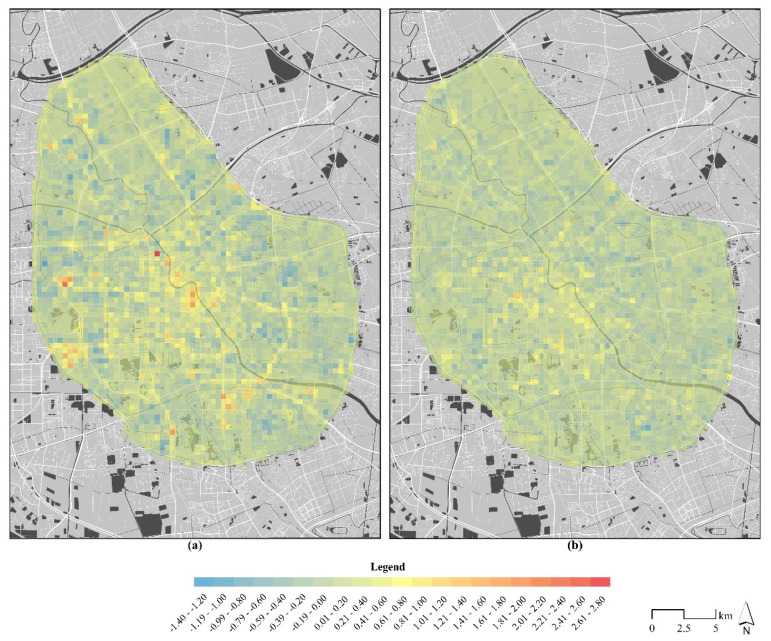
The population tidal index distribution during the pre-epidemic (**a**) and epidemic (**b**) periods.

The tidal intensity distribution results of the day-and-night population flows during the pre-epidemic and epidemic periods are shown in Figure 4. The red color indicates that the daytime population aggregation level was higher than the nighttime level, while the blue color has the opposite meaning. High-value units of tidal intensity during the pre-epidemic period were commonly found along the Haihe River and main roads. While during the epidemic period, the tidal intensity in the study area was reduced from 4.85 to 2.25, and the maximum values occurred around hospitals (Nankai Hospital and Tianjin Maternity Hospital). The strong tidal flow along the Haihe River disappeared, and the spatial characteristics indicated a homogenization in the study area.

### 3.4. Aggregation Characteristics of the Urban Population Tidal Intensity

The tidal-intensity Moran’s I index value for the pre-epidemic and epidemic periods were 0.323 and 0.209, respectively. The pre-epidemic period displayed a more obvious population aggregation than the epidemic period.

**Figure 5:**
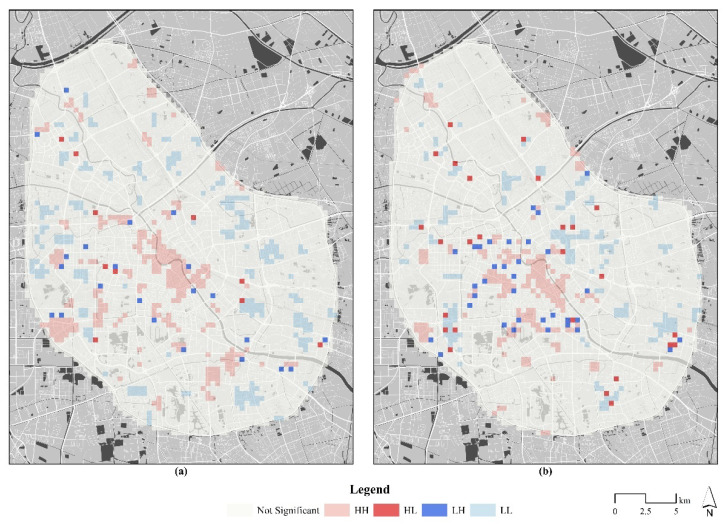
The local Moran’s I results during the pre-epidemic (**a**) and epidemic (**b**) periods.

We used the local Moran’s I to distinguish the spatial aggregation types, and HH (clustered population inflow), LL (clustered population inflow), LH (local population inflow), and HL (local population outflow) units were identified (Figure 5). During the pre-epidemic period, the HH type appeared mainly around the middle section of the Haihe River and the Huayuan Residential District. The LL type was distributed mainly on the edge of the study area, and there were a few units of the LH and HL types surrounding peripheral small HH and LL patches. During the epidemic period, the number of HH type units decreased and a fragmented pattern developed, with the number of LL types increasing and occurring mainly in the core area. The numbers of LH type units and HL type units increased significantly and were spatially interspersed with HH and LL units.

During the pre-epidemic period, the study area presented a stable single-core of the work-residential structure with more long-distance commuting and less local population flows. The study area constituted a large living circle. During the epidemic period, travel demand was reduced, long-distance travel was restricted, and the main population outflow units were split into many smaller population outflow patches. These patches were also surrounded by local population inflow units, indicating that short-distance travel had become the main population mobility trend. The urban living area was separated into several small local clusters. Urban living units were dispersed into several small units, and the residential community was closer to the surrounding functional spaces.

**Figure 6:**
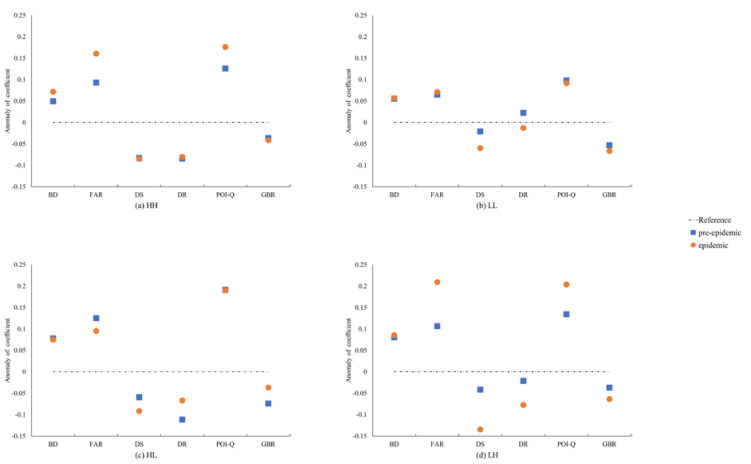
Anomalies of factors’ coefficient during the pre-epidemic and epidemic periods. (**a**) HH type; (**b**) LL type; (**c**) HL type; (**d**) LH type.

We calculated the anomalies in the coefficients during the pre-epidemic and epidemic periods to determine the differences in population-flow characteristics (Figure 6). Indicators with large differences between the pre-epidemic and epidemic periods (e.g., FAR and POI-Q as shown in Figure 6a) were the main factors responsible for the change in HH clusters during the different periods. Pre-epidemic HH units were composed mostly of office buildings, business, parks, and factories, and had a high BD and small FAR. The proportion of these spaces was reduced in the epidemic period HH units. The average FAR and POI-Q increased because of the increase in people remaining in residential communities. In contrast, indicators in which there was less difference (e.g., DS and DR), had very little influence in HH units. The LL units represented the typical living space in the city, as shown in Figure 6b. There were few differences in BD, FAR, POI-Q and BGR between the pre-epidemic and epidemic period. The road accessibility index (DS, DR) decreased significantly in the epidemic period. During the epidemic, the work-life balance of urban residents was weakened, and travel intensity decreased, resulting in the decreases in the relevant indicators. The BD and POI-Q of HL units decreased and the DR and BGR increased, as shown in Figure 6c. The centripetality of the HL type units was enhanced. Most of these units were service facilities in local residential areas and were located adjacent to LL units, satisfying most of the short-distance shopping demand during the epidemic. This type of unit had little demand of location and public transportation, but it needed the convenient road traffic and space quality. In Figure 6d, the anomalies of FAR and POI-Q of LH units increased, and the traffic accessibility index decreased significantly. Most LH units were located adjacent to HH units, indicating the significance of short travel distances in work-centered residential areas. This was caused by the impact of the reduction in demand for shopping and entertainment. This type of unit developed by the gathering of peripheral HH patches to all HH clusters, verifying the fragmentation of the urban lifestyle from the pre-epidemic to epidemic periods.

## 4. Discussion

Population distribution and its tidal characteristics are the most important expressions of local genes. They are the outcomes of differences in the spatial characteristics of urban material space, functional space and public policy.

While faced with a public-health emergency, the use of material space will undergo a subversive transformation. Demand for outdoor activities transforms to demand for home-based protection; leisure space, shopping, and entertainment spaces are closed; public-health facilities are over-operating; and the road utilization rate greatly decreases. These phenomena will sever the links between urban functional space units. Low-activity space is used to improve urban resilience. Based on the distribution characteristics and daytime-nighttime tide of urban population during pre-epidemic and epidemic periods, this study investigated the temporal differences in use of urban space and the results will help to improve the resilience of urban public-health measures.

### 4.1. Implications for Public-Health Resilience in High Density Urban Areas

In China, the reconstruction of material space use in response to the COVID-19 outbreak has become a new topic in the field of urban research. Many studies have investigated regional urban connections with inter-city population flows, but the daily spatial behavior of residents has rarely been considered. This study bridged this gap by comparing the demographic spatial distribution during the pre-epidemic and epidemic periods and evaluated its tidal characteristics as well.

Urban spatial vitality has been the main focus of urban planning and construction, but the public-health emergency completely reversed this trend. There was a need for novel spatial control strategies that encouraged minimal contact among the population when the health emergency occurred. This included the shutting down schools, shopping malls, and other functional places, while allowing for the over-running of hospitals, community neighborhood committees, and governments. The lifestyle changed from the pursuit of a high-quality lifestyle to a basic living security under the minimum travel time and distance. The intensity of population, information and material flows decreased substantially, and the dependence on transport also decreased, while the demand for convenient services increased. The community’s external connections were weakened, and some travel networks were subject to controls. Mapping to population distribution, people in leisure, tourism, and shopping spaces was substantially decreased, particularly in locations along the Haihe River. Aggregation decreased in the units next to some important roads (e.g., Heiniucheng Road and Kunlun Road). It confirmed that the work-life balance was broken, and the population aggregation in residential areas increased considerably.

This study provides reference for future urban epidemic prevention and protection, and urban spatial control during a public-health emergency. Adjacent areas of HH-LH units constituted a spatial mode for the gathering of the daytime population and nighttime population outflows. In these units, evaluating the risk of regional exposure in real time and monitoring the movement trajectory of vulnerable populations were important to improve the relevance and effectiveness of epidemic surveillance. Adjacent areas of LL-HL units formed a community-centered local living circle. Unlike the pre-epidemic period, short-distance living demands became the driving force of this population distribution pattern.

**Figure 7:**
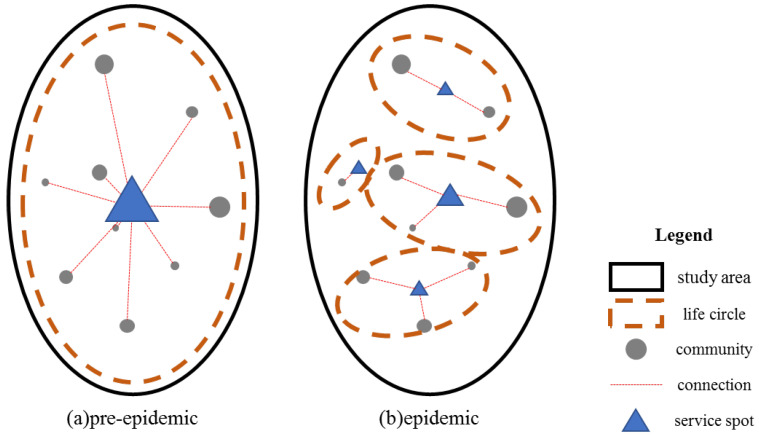
Living-circle modes of the pre-epidemic (**a**) and epidemic (**b**) periods.

Before the public-health emergency arose, the urban space could be considered similar to a large living circle, with a high degree of integration (Figure 7a). The city center had the highest service level, while urban residents lived in the periphery. Their aim was to pursue a high quality of life and functional diversity in high-density areas of the city. The public-health emergency interrupted the connections within the city area and divided the large living circle into several basic living units (Figure 7b), in which the residents’ needs for basic living were satisfied in areas closer to their place of residence. This shows the form of urban spatial organization that will ultimately be required to safeguard public health. The most important feature of resilience in urban public health is to satisfy the supply needs of the basic living circle units, including grain, oil, and medical protective equipment. It is necessary to build a service system for epidemic prevention and control units.

### 4.2. Limitations of the Study

This study clearly revealed the differences in population distribution and day-night tidal characteristics during different periods in the core urban area of Tianjin, but there were still some limitations. First, BHM is an important data source for population aggregation evaluation that can effectively reflect the real-time urban population distribution. It is a type of hierarchical raster numerical data, which is calculated by a certain algorithm that is generated based on the number of terminal access points. Therefore, it is not able to visually present the absolute number of residents, but can consider only the differences in the relative numbers of residents in the unit through a grade assignment. This has a large influence on the accuracy of population distribution. Second, different groups of people have different life characteristics [50,51]. In this study, it was difficult to determine the differences among different groups, such as old people, women, and children.

In the future, the use of multi-source data would help to optimize the method to determine the population spatialization and to obtain more details for the different groups. Improvement of population distribution data accuracy should also be a focus of future research, which would greatly improve the accuracy of future studies.

## 5. Conclusions

By comparing the urban population distribution and daytime-nighttime tides during pre-epidemic and epidemic periods, this study analyzed the differences in population distribution before and during a public-health emergency, and figured out its controlling and organizational mechanisms. The study quantitatively described the heterogeneity of urban space and reached several key conclusions.

Epidemic population density decreased in the central area, and the single core circle distribution pattern dissolved partly. The decreasing demand for open spaces and transportation and the increasing demand for indoor activity, resulted in a decline in population aggregation in high-accessibility areas. This was a consequence of the enhanced urban space control and the self-control of residents when facing the risk of infectious diseases. These actions led to a reduction in the frequency of medium/long-distance travel. The need is obvious that to regress to a less expansive lifestyle, and an increase in the amount of home-based activities. Because the POI-Q and BGR are subsidiary indexes within the scope of a change in the living area, they had a weak impact and presented little difference between the pre-epidemic and epidemic periods.

The decrease in the population tidal intensity indicated a decrease of population flow. The spatial characteristics of the population tides tended to be homogeneous. There were four types of tidal aggregation in the results: HH, LL, LH and HL. It was found that the pre-epidemic city presented a large living circle spatial pattern, with spatial distance having little impact on an individual’s willingness to travel, and residents tended to pursue a high-quality lifestyle. During the epidemic period, the living circle was limited by travel capacity and willingness. It transformed into several local living circles. Basic living security facilities around the community became an important component of the local living circles and their importance was greatly enhanced. The overall living circle in high-density areas was greatly weakened, and serval smaller basic living units emerged. During a public-health emergency, we should attempt to improve support measures for there basic units and control the flow intensity of the overall living circle.

Material space, functional space, and public policy influenced the city in different ways and with different intensities during the pre-epidemic and epidemic periods. They led to a substantially different population distribution. This study proposed the need of different urban spatial response strategies under different situations. During pre-epidemic periods, increasing the spatial function attraction and traffic accessibility will lead to population concentrations in small areas, improving local spatial vitality. During epidemic periods, organizing basic living units, controlling the safety threshold of population aggregation, and configuring basic public service facilities will guarantee the convenience of residential life.

## Figures and Tables

**Table 1:** The GWR model results for the pre-epidemic period.

Variable	MEAN	STD	MIN	MAX	Area Ratio of Significance (%) *	+	-
Intercept	0.228	0.097	−0.015	0.529	90.89%	100.00%	0.00%
BD	−0.241	0.198	−1.051	0.394	37.55%	0.00%	100.00%
FAR	0.401	0.243	−0.126	1.789	46.04%	100.00%	0.00%
DS	−0.089	0.203	−1.017	0.359	13.83%	81.93%	18.07%
DR	−0.305	0.18	−1.001	0.032	69.09%	0.00%	100.00%
POI-Q	0.533	0.162	0.015	1.098	93.75%	100.00%	0.00%
GBR	−0.12	0.179	−0.873	0.718	12.77%	0.00%	100.00%
RSS	29.468						
sigma	0.09						
AICc	−6419.5						
R^2^	0.792						
Adjusted R^2^	0.766						

* Area ratio of significance is the proportion of significant (*p* < 0.10) grids in the study area.

**Table 2:** The GWR model results for the epidemic period.

Variable	MEAN	STD	MIN	MAX	Area Ratio of Significance (%) *	+	−
Intercept	0.143	0.08	−0.082	0.475	60.37%	100.00%	0
BD	−0.304	0.246	−1.084	0.522	42.88%	0	100.00%
FAR	0.669	0.398	−0.788	1.608	71.09%	99.96%	0.04%
DS	−0.057	0.227	−0.993	0.531	13.50%	34.36%	65.64%
DR	−0.082	0.135	−0.793	0.359	8.28%	8.39%	91.61%
POI-Q	0.543	0.253	−0.059	1.472	83.92%	100.00%	0
GBR	−0.139	0.208	−1.19	0.681	9.16%	2.73%	97.27%
RSS	38.927						
sigma	0.104						
AICc	−5417.107						
R^2^	0.753						
Adjusted R^2^	0.722						

* Area ratio of significance is the proportion of significant (*p* < 0.10) grids in the study area.

## Data Availability

No new data were created or analyzed in this study. Data sharing is not applicable to this article.
